# 

**Published:** 1874-05

**Authors:** 


### THE AMERICAN JOURNAL OF DENTAL SCIENCE,

EDITED BY

F. J. S. GORGAS, M. D., D. D. S.

VOL. 8.—THIRD SERIES.

1874-5.

Surgeon Genl's Office
LIBRARY.
W63714
Washington, D.C.

BALTIMORE:

SNOWDEN & COWMAN.

LONDON:

TRUBNER & CO., 60 PATERNOSTER ROW.

1875.

### INDEX TO VOLUME VIII.---THIRD SERIES.

Advantage of Capping Pulps of Teeth, - - - - 20
Administration of Ether, - - - - - - - - 46
Apparent Death. Recognition by Faradization, - 44
Anæsthetization During Sleep, - - - - 42, 285
Alcohol in Medical Practice, - - - - - - - 58
Agassiz Memorial, - - - - - - - - - 91
Arsenious Acid, Physiological Action of, - - - 423
A New Sign of Death, - - - - - - - - 95
A Dentist Fined, - - - - - - - - 96
A New Dental Hospital, - - - - - - - 96
A New Method of Extracting Temporary Teeth, - 183
A French Charlatan, - - - - - - - 190
A Safe and Simple Method for Self Administration of
Chloroform, - - - - - - - 278
A Little Studied Cause of Hoarseness, - - - - 45
A Lost Case, - - - - - - - - - 333
A Warning to Dentists, - - - - - - 427
A Toothless People, - - - - - - - 472
A Simple Plan of Ventilation, - - - - - - 476
American Dental Association, - 139, 276, 347, 395, 445
American Dental Convention, - - - - 120, 139
American Academy of Dental Science, - - - 139
American Dental Society of Europe, - - - - - 139
Austen, Prof. P. H., M. A., M. D., D. D. S., - - 145
Action of Different Medicines used in Dental Practice, 261
Amalgams, On - - - - - - - - - 270
Annual Address before the American Academy of Dental
Science, - - - - - - - - 289
Allport, W. W., D. D. S., M. D. - - - - - - 289
Arthur's Treatment, Dr. C. S. Smith on - - - - 344
Arsenic Poisoning by a Green Carpet, - - - - 381
Anæsthesia, - - - - - - - - - 385
Ante-natal Development of Nine Teeth, - - - 432
Atrophy of the Teeth. - - - - - - - 440

Anæsthesia by Injection of Chloral into the Veins, - - 479
An Unexpected Property of Adhesive Gold, - - 29
American Medical Degrees - - - - - - - - 480
Abnormal Mucous Membrane, - - - - 121
Address by W. W. H. Thackston, M. D., D. D. S - 481
An Insecticide, - - - - - - - 525
Amalgams for the Teeth, - - - - - - 558
An Unnatural Position of the Head a Cause of Death from Anæsthetics, - - - - - - 570
A New Operation for Cleft Palate, - - - - 571
An Extraordinary Case, - - - - - - 573
Acidity of the Gastric Juice - - - - - - 574
Actual Cautery, - - - - - - - - 574
Assaying by the Spectroscope, - - - - - - 575
Baltimore College of Dental Surgery, Thirty-fifth Annual Session of - - - - - - - - 325
Baltimore College of Dental Surgery, Annual Commencement - - - - - - - - - 470
Black, G. V., D. D. S., - - - - - - - 1
Bichloride of Methylene, Use and Merits of - - 157
Brown, Sequard, M. D., - - - - - - - 82
Brain Power of Man, - - - - - - 82
Bromide of Potassium in Convulsions and Dental Irritation, 89
Bonnell, W. G. A, D. D. S., - - - - - 129
Blue Pus, - - - - - - - - - 143
Burgh, Thos., D. D. S., - - - - - - 176
Bone and Skin Grafting, - - - - - - - 191
Bogue, E. A., D. D. S., - - - - - - 279, 558
Buck, Gurdon, M. D.. - - - - - - - 369
Bunnell, Dr. E. F., - - - - - - - 385
Burkholder. N., D. D. S., - - - - - - 440
BIBLIOGRAPHICAL.
Physiology of Man, - - - - - - 235
Chemical Lectures on Diseases of Nervous System, 330
Instructions in Manipulation of Vulcanite, - 330
Physician's Visiting List for 1875, - - - 330
Capping Pulps of Teeth, Advantage of - - - - 20
Can a Person be Anæsthetized During Sleep ? - - 42
Cancer Cured by Faith, - - - - - - 46

Cure for Headache, - - - - - - - 48
Cobb, S. J., D. D. S., - - - - - - - 548
Contour Fillings, The Practical value of - - - 67
Cremation. - - - - - - - - - 87, 334
Carbolic Acid in Carbuncle, - - - - - - 92
Chloral Hydrate and Camphor in Neuralgia, - - 94
Care of Children's Teeth Between Ages of Six and Fifteen, 105
Causes of Decay of the Teeth, - - - - - 133
Cleft Palate, New Operation for - - - - - 135
Chloral as a Preservative, - - - - - - 526
Chloral Poisoning Cured by Strychnia, - - - 142
Chlorate of Potash in Cancer, - - - - - - 287
Case of Resuscitation from Apparent Death from Chloroform, - - - - - - - - - 505
Causes of Constitutional Derangement in First Dentition, Principal - - - - - - - - 165
Causes of Death from Petroleum Explosion, - - 575
Comments upon Dr. Allport's Address, - - - 506
Copeland, G. W , M. D., - - - - - - 180
Caries, Ætiology of Dental - - - - - 241
Changes in the Shapes of Teeth that are Proper for Treatment of Decay, - - - - - - 250
Chase, Henry S , M. D., D. D. S., - - - - 256, 464
Carvacrol, - - - - - - - - - - 279
Camphorated Phenol, - - - - - - - 284
Chlorate of Potash in Cancer, - - - - - 287
Chloroform Tippler, - - - - - - - - - 287
Connection of Cancer and Skin Disease, - - - 331
Coles, Oakley, L. D. S., - - - - - - 361, 410
Class Address, - - - - - - - - - 542
Chloral Hydrate and Camphor in Neuralgia, - - 572
Celluloid, - - - - - - - - - 376, 432
Condition of the Mouth and Teeth during Pregnancy, 361, 410
Cicatricial Contraction from Burns Involving the Face, 369
Cure for Toothache, - - - - - - - - - 239
Cause of so many Failures, - - - - - - 376
Chloral, Local Use of - - - - - - - 378
Cutting Teeth, - - - - - - - - - 378
Cushing, George H., D. D. S., - - - - - 216

Correction, - - - - - - - - 472
Colored Inks, - - - - - - - 477
Danger of Chloroform After Chloral, - - - 525
Dental Pathology and Surgery, - - - - 521
Decision Affecting Dentistry, - - - - - 528
Dentistry, - - - - - - - - 145
Diseased Conditions, Their Effects Upon the Teeth, - 1
Diarrhœa of Teething Children, - - - - 40
Death from Chloroform, - - - - - 45, 382
Disease Destroying Tree, - - - - - - 93
Death from Chloroform and Hemorrhage - - - 96
Determination of the Age of the Human Embryo by the
Evolution of the Teeth, - - - - - 144
Devitalizing Dental Pulps, The most Efficacious Form of
Arsenic for - - - - - - - 176
Dawson, Benj. F., M. D., - - - - - - 157
Death from a Dose of Chloral and Bromide of Potassium, 190
Death from Lancing Gums, - - - - - - 191
Dental Caries, Ætiology of - - - - - 241
Dental Pulps, Conservative Treatment of - - 224
Defective Palate, - - - - - - - 329
Danger of Mixing Chromic Acid with Glycerine, - 335
Directions for Escaping Colds, - - - - - 384
Dental Operations, Thoroughness and Honesty in - 433
Difference in the Composition of Teeth, - - 576
Diseases of the Enamel, - - - - - - 464
Dentes Sapientiæ - - - - - - - - 471
Disinfectants, - - - - - - - - - 476
Ætiology of Dental Caries, - - - - - 241
Ether and Chloroform, - - - - - - 239
Extraordinary Malpractice, - - - - - - 94
Efficacious Form of Arsenic for Devitalizing Pulps, - 176
Extracting Temporary Teeth, A New Method of - 183
Excision of the Superior Maxillary, - - - - 324
Epithelial Cancer of the Tongue, - - - - 337
Experiments Upon the Human Brain - - - 237
East Tennessee Dental Association, - - - 345
Electric Light, - - - - - - - - 572
Electricity a Substitute for Gas or Coal Oil, - - 431

Electricity in Vomiting, - - - - - - - 382
Electricity for Chilblains, - - - - - - 239
Extraction of Teeth, - - - - - - - 390
Enamel, Diseases of - - - - - - - 464
Eubank, A., D. D. S., - - - - - - 505
Extensive Destruction of Soft Parts of Mouth from
Sloughing after Fever, - - - - - - 468
External Application of Iodine, - - - - 478
Effects of Inferior Teeth Upon Alveolar Process of
Snperior Maxillary, - - - - - - 329
Exostosed Teeth, - - - - - - - - 329
Eucalyptus and Tobacco, - - - - - - 480
Fairbank, John, M. R. C. S., - - - - - - 37
False Tongue, - - - - - - - - 525
Fletcher, Thomas, F. C. S. - - - - - 29
Fine Wire, - - - - - - - - - 572
French Charlatan, - - - - - - - - 180
Fillings, Plastic - - - - - - - - 282
Finlayson, Dr. James, - - - - - - - 516
Fouke, Dr. George S., - - - - - 16, 153, 222
Gelseminum in Odontalgia, - - - - - - 332
Godfrey, Robert, M. D., - - - - - - 468
Guarana for Cure of Headache, - - - - 48
Handy Method of Examining Nervous Tissues Microsco-
pically, - - - - - - - - 432
Hardman, Dr. J. - - - - - - - - 169
Headache from Eye Strain, - - - - - 575
Hygiene of Dwellings, - - - - - - - 48
Harlan, Dr. A. W. - - - - - - - - 241
Howard, Dr. F. E. - - - - - - - - 20
How to Remove Rings from the Finger, - - - - 477
How New York Physicians and Dentists get a Competence, 89
How to Pull Teeth, - - - - - - - 332
How to Check Coughs, - - - - - - - 144
How to Deprive Iodine of its Stain, - - - - 381
How to Use Plastic Fillings, - - - - - - 282
How to Insert the Hypodermic Syringe, - - - 380
Hours of Maximum Mortality in Acute and Chronic
Diseases, - - - - - - - - 287

Hubbard, Dr. - - - - - - - - 337
Hydrogen Alloys, - - - - - - - - 576
Improved Method of Packing Moulds, - - - 222
Impressions of the Mouth, Taking Upper - - 16, 153
Improvements in Dentistry, - - - - : - 32
Iodoform as a Topical Remedy, - - - - - 189
Iodine and Preparations, Mode of Action of - - 171
Ionic Acid, Use of, Hypodermically, - - - - 190
Influence of Fear in the Production of Disease, - 286
Influence of Anæsthetics upon the Sexual Impressions of Females, - - - - - - - 431
Injury of the Face, - - - - - - - 564
Intra-Buccal Resection of Inferior Dental Nerve, - 428
Keller, D., M. D., - - - - - - - - 324
King, James M., D. D. S. - - - - - - 542
Kirby, Dr. Amos - - - - - - - - 279
Kulp, W. O., D. D. S., - - - - - - - 64
Large Calculus, - - - - - - - - 188
Lacto-phosphate of Lime for Carious Teeth, - - 576
Latimer, Thomas S., M. D., - - - - - - 526
Laughter as a Medicine, - - - - - - - 47
Lee, Professor - - - - - - - - 171
Lusus Naturæ, - - - - - - - - - 191
Large Fees, - - - - - - - - - 234
Lane, W. H., M. D., - - - - - - - 278
Lancing the Gums, - - - - - - 383, 516
Longevity of Medical Men, - - - - - - 334
Local Use of Chloral, - - - - - - - 378
Lock Jaw and Quinia, - - - - - - - 527
Lead as a Constituent of Enamel the Cause of Chronic Poisoning, - - - - - - - 380
Lymphatic Tumors of the Face, - - - - - 574
Manufacture of Caoutchouc from Milk Weed, - 571
Mechanical Dentistry, - - - - - - - 64
Marine Glue, - - - - - - - - - 239
Monstrosity, - - - - - - - - 96
Morris, John, M. D., - - - - - - - 58, 113
Mode of Action of Iodine and Preparations. - - 171
Methyl, Bichloride - - - - - - - 191

Monobromide of Camphor, - - - - - 381
Moth Preventive, - - - - - - - - 476
Malignant Pustule from a Fly-bite, - - - - 527
Murray, Dr. Jno. - - - - - - - - 261
Noel, Professor Henry R., M. D,, - - - - - 49, 97
New Operation for Cleft Palate, - - - - - 135
Neuralgia Treated by Phosphorous, - - - - 141
New Method of Extracting Temporary Teeth, - - 183
New Sign of Death, - - - - - - - 525
Nickeling - - - - - - - - - - 187
Noyes, Dr. Edmund, - - - - - - - 250
Nothing New Under the Sun, - - - - - 383
Neuralgia of Lower Jaw and Tongue Treated by Excision of the Nerve. - - - - - - - 474
Nelaton's Method of Resuscitation from Chloroform Narcosis, - - - - - - - 478
New Operation for Cleft Palate and Blind Uvula, - 527
Operative Dentistry, - - - - - - - 548
Oxygen Gas as a Remedy in Disease, - - - - 144
On Mode of Action of Iodine, - - - - - - 171
On Amalgams, - - - - - - - - - 270
Oleate of Mercury in Ringworm, - - - - - 333
On Condition of Mouth and Teeth During Pregnancy, 361, 410
OBITUARY.
T. B. Hitchcock, D. D. S., M. D., - - - - - 234
George E. Hawes, D. D. S., - - - - - - 377
Eleazar Parmly, M. D., D. D. S., - - - - 522
Asa Hill, D. D. S., - - - - - - - - 523
James Fleming, M. D., D. D. S., - - - - 524
T. B. Hamlin, - - - - - - - - - 140
Protoplasm, Its Relation to Vitality, - - - - 49, 97
Poisoning by Cantharidal Collodion, - - - - - - 95
Principal Causes of Constitutional Derangement in First Dentition, - - - - - - - - 165
Poisoning from Amalgam Fllings, - - - - - 184
Part which Vital Action Plays in the History of Dental Caries, - - - - - - - - 256
Position of Women, - - - - - - - - 286
Preservation of Leeches, - - - - - - - 526

Prevention of Cicatrices, - - - - - - 190
Practice of Dentistry in South Carolina, - - 567
Proceedings of American Dental Association, - 139, 305, 347
Proceedings of Virginia Dental Association, - 495, 553
Proceedings of Georgia State Dental Society, - - 503
Precautions Against Trichina, - - - - 528
Prevention of Dental Caries, - - - - - - 381
Physical Peculiarities of Negroes, - - - - 382
Physiological Action of Arsenious Acid, - - - 423
Preparing Vegetable Tissues for the Microscope, - 427
Passage of Arsenic and Antimony into the Tissues and
Secretions, - - - - - - - - 478
Precautions During Anæsthesia, - - - - - 514
Preservation from Hydrophobia, - - - - 237
Remedy for Toothache, - - - - - - 95, 189
Root Filling, - - - - - - - - - 35
Replantation of Teeth, - - - - - - - 41
Remedy for Chronic Hoarseness, - - - - - 46
Recent Decision in the Vulcanite Suit, - - - 136
Resection of Upper Jaw, with Preservation of Portion
of Hard Palate and Incisor Teeth, - - - 569
Report of Committee on Mechanical Dentistry, - - 301
Rubber Dam, - - - - - - - - - 318
Richardson, J., D. D. S., - - - - - - 506
Richardson's Tooth-edge Cutting Scissors, - - - 475
Saliva, Record of Tests of - - - - - - 216
Separating Teeth, - - - - - - - - 129
Successful Case of Transfusion, - - - - 96
Spalding, C. W., D. D. S., - - - - - 35
Southern Dental Association, Proceedings of - 138, 193, 425
South Carolina Dental Association, - - - - 182
Solubility of Arsenious Acid in Alcohol, - - 186
Styloid Muscles and Anæsthetics, . - - - 180
School Diseases, - - - - - - - - 283
Spongoid, - - - - - - - - - 326
Salivary Calculus, - - - - - - 329, 419
Smith, C. S., D. D. S., on Arthur's Treatment, - - 344
Supplemental Nerve Force, - - - - - - 524
Salter, James A., F. R. S., - - - - - - 419

Salivary Fistula, - - - - - - - - 474
Sage, H. L., D. D. S. - - - - - - 67, 224
Singular Death from Blood Poisoning, - - - - 239
Taking Upper Impressions of the Mouth, - - 16, 153
Tenotomy in Treatment of Fracture of Lower Jaw, - 575
Thurman, J. S., M. D., - - - - - - - 564
Treatment of Dog Bites, - - - - - - - 429
Treatment of Chloroform Asphyxiation, - - 45
Treatment of Malignant Pustule, - - - - - 430
Treatment of Salivation by Atropia, - - - - 46
Treatment of Wounds, - - - - - - - 473
Treatment of Epistaxis, - - - - - 525
Treatment of Mucous Polypus of Velum Palati - 562
The Medical Education of Women, - - - - 334
To Suppress Hemorrhage of the Mouth During Operations, 334
The Areca or Betel Nut, - - - - - - 47
Trizzard, S. B., M. D., - - - - - - - 121
Trichinæ, - - - - - - - - - - 191
The Cause of so Many Failures, - - - - - 376
To Arrest Hemorrhage of Mouth and Gums, - 383
To Keep Away Flies, - - - - - - - 431
Thoroughness and Honesty in Dental Operations, - 433
Townsend, Dr. H. H., - - - - - - - 433
The St. Louis Meeting, - - - - - - - 432
The Thirty-Fifth Annual Commencement of the Baltimore College of Dental Surgery, - - - 518
Treatment of Poisoning by Chloral, - - - - - 238
Use of Alcohol in Medical Practice, - - - - 58, 113
Use and Merits of Bichloride of Methylene as an Anæsthetic 157
Use of Iodic Acid by Hypodermic Injection, - - 190
Useful Hints, - - - - - - - - 233
Valedictory Address, - - - - - - - 526
Virginia State Dental Association, - - - - - 375
Vulcanite Litigation, - - - - - - - 280
Weight of Men and Women, - - - - - - 479
Zinci Oxychloridum, - - - - - - - - 37

### TO READERS AND CORRESPONDENTS.

Communications intended for publication, and exchange journals and papers, should be addressed to the EDITOR OF THE AMERICAN JOURNAL OF DENTAL SCIENCE, 259 N. Eutaw St., Baltimore, Md. All letters relating to the business of the journal, or containing cash remittances, to be directed to Messrs. SNOWDEN & COWMAN. Articles for publication should be received by the editor at least three weeks previous to the first day of the month on which the number in which it is designed for them to appear, is issued.

### CONTENTS:

### ORIGINAL COMMUNICATIONS.

Replantation of Teeth.................................... 41

Can a Person be Anæsthetized During Sleep?.................................... 42

### MONTHLY SUMMARY.

Apparent Death—Recognition by Faradization.................................... 44

Death from Chloroform—Damages.................................... 45

Treatment of Chloroform Asphyxiation.................................... 45

A Little Studied Cause of Hoarseness.................................... 45

Treatment of Salivation by Atropia.................................... 46

Cancer Cured by Faith.................................... 46

Remedy for Chronic Hoarseness.................................... 46

Administration of Ether.................................... 46

The Areca or Betel Nut.................................... 47

Laughter as a Medicine.................................... 47

Hygiene of Dwellings.................................... 48

Guarana for Cure of Headache.................................... 48

☞The American Journal of Dental Science will contain fifty pages of reading matter in each number, making a volume of not less than

*Six Hundred* pages

EXCLUSIVE OF ADVERTISEMENTS.

THE

### AMERICAN JOURNAL

OF

### DENTAL SCIENCE.

**F. J. S. GORGAS, M. D., D. D. S., Editor.**

The *Journal* is issued on the first of each month and contains not less than *fifty* pages of reading matter in each number, exclusive of advertisements, making a volume of *six hundred* pages per annum.

In each number will be found Original Communications, Correspondence, Selected Articles, Monthly Summary, Bibliographical Notices and Editorial Matter, and every effort will be exerted to render the *Journal* worthy of the patronage of the Dental profession.

A generous co-operation is respectfully solicited from the members of the profession; and as the *Journal* has now a printing office of its own which materially lessens the cost of its publication, it will be issued at the reduced subscription price of

**$2.50 Per Annum in Advance.**

Communications are respectfully solicited on all subjects pertaining to the practice of dentistry, as well as reports of cases occurring in dental practice.

### RATES OF ADVERTISING.

| | 1 MO. | 2 MO. | 3 MO. | 6 MO. | 12 MO. |
|---|---|---|---|---|---|
| 1 PAGE. | $15 00 | $22 50 | $30 00 | $50 00 | $100 00 |
| ½ " | 10 00 | 15 00 | 20 00 | 30 00 | 50 00 |
| ¼ " | 7 00 | 11 00 | 15 00 | 20 00 | 30 00 |
| ⅛ " | 5 00 | 8 00 | 11 00 | 15 00 | 20 00 |

All original communications designed for publication, works for review, new instruments for notice, exchanges, &c., should be directed to the editor, 259 N. Eutaw St., Baltimore, Md.

All advertisements and subscriptions should be addressed to

SNOWDEN & COWMAN,

*Publishers of the Am. Journal of Dental Science.*

86 W. FAYETTE ST. BET. CHARLES & LIBERTY STS.,

BALTIMORE, Md.

